# ABCA8 is regulated by miR-374b-5p and inhibits proliferation and metastasis of hepatocellular carcinoma through the ERK/ZEB1 pathway

**DOI:** 10.1186/s13046-020-01591-1

**Published:** 2020-05-19

**Authors:** Yifeng Cui, Shuhang Liang, Shugeng Zhang, Congyi Zhang, Yunzheng Zhao, Dehai Wu, Jiabei Wang, Ruipeng Song, Jizhou Wang, Dalong Yin, Yao Liu, Shangha Pan, Xirui Liu, Yan Wang, Jihua Han, Fanzheng Meng, Bo Zhang, Hongrui Guo, Zhaoyang Lu, Lianxin Liu

**Affiliations:** 1 Department of Hepatic Surgery, The First Affiliated Hospital of Harbin Medical University, Harbin, Heilongjiang China; 2 Key Laboratory of Hepatosplenic Surgery, Ministry of Education, Harbin, Heilongjiang China; 3 Department of Hepatobiliary Surgery, The First Affiliated Hospital of University of Science and Technology of China, Hefei, Anhui China; 4 Department of Colorectal Surgery, Harbin Medical University Cancer Hospital, Harbin, Heilongjiang China

**Keywords:** Hepatocellular carcinoma, ATP binding cassette subfamily a member 8, Epithelial to mesenchymal transition, Therapeutic target

## Abstract

**Background:**

ATP binding cassette subfamily A member 8 (ABCA8) belongs to the ATP binding cassette (ABC) transporter superfamily. ABCA8 is a transmembrane transporter responsible for the transport of organics, such as cholesterol, and drug efflux. Some members of the ABC subfamily, such as ABCA1, may inhibit cancer development. However, the mechanism of ABCA8 in the process of cancer activation is still ambiguous.

**Methods:**

The expression of ABCA8 in human hepatocellular carcinoma (HCC) tissues and cell lines was examined using qPCR, immunoblotting, and immunohistochemical staining. The effects of ABCA8 on the proliferation and metastasis of HCC were examined using in vitro and in vivo functional tests. A luciferase reporter assay was performed to explore the binding between microRNA-374b-5p (miR-374b-5p) and the ABCA8 3′-untranslated region (UTR).

**Results:**

ABCA8 was frequently down-regulated in HCC and this down-regulation was negatively correlated with prognosis. The overexpression of ABCA8 inhibited growth and metastasis in HCC, whereas the knockdown of ABCA8 exerted the antithetical effects both in vivo and in vitro. ABCA8 was down-regulated by miR-374b-5p; this down-regulation can induce epithelial transformation to mesenchyme via the ERK/ZEB1 signaling pathway and promote HCC progression.

**Conclusion:**

We exposed the prognostic value of ABCA8 in HCC, and illuminated a novel pathway in ABCA8-regulated inhibition of HCC tumorigenesis and metastasis. These findings may lead to a new targeted therapy for HCC through the regulation of ABCA8, and miR-374b-5p.

## Background

Hepatocellular carcinoma (HCC) accounts for the majority of primary liver cancer, ranks as the sixth most common cancer, and was the fourth leading cause of cancer death worldwide in 2018 [1]. The main pathogenic factors of HCC are viral hepatitis infection (HBV/HCV), ingestion of aflatoxin, diabetes, tobacco intake, and heavy alcohol intake [2–4]. Surgical treatment is an effective treatment for liver cancer, but recurrence and metastasis may still occur, limiting the overall survival of HCC patients [5]. Therefore, further understanding of the molecular mechanisms related to HCC can help us determine effective therapies to combat the recurrence and metastasis of HCC.

ATP binding cassette subfamily A member 8 (ABCA8) is a member of the ABC transporter superfamily, of which, human beings have 48 transcriptionally active ABC transporter genes divided into 7 subfamilies, A-G [6]. ABCA1 and ABCA8 are homologous and belong to the same subfamily. ABCA1 is believed to inhibit the proliferation and metastasis of many cancers [7–9]. However, the role of ABCA8 in tumorigenesis and the mechanism by which ABCA8 acts remain unclear, particularly in HCC.

In this study, we used clinical data and molecular biological experiments to clarify the mechanism by which ABCA8 impacts HCC. The results showed that ABCA8 was frequently down-regulated in HCC and that decreased ABCA8 was associated with poor prognosis, tumorigenesis, and metastasis. ABCA8 also proved to be down-regulated by miR-374b-5p, which in turn was up-regulated in HCC and resulted in the progression of HCC via the ABCA8/ERK/ZEB1 signal pathway.

## Methods

### HCC specimens

We collected matched HCC and adjacent non-tumor tissue from patients who underwent hepatectomy in the First Affiliated Hospital of Harbin Medical University between August 2010 and September 2014. We invited senior pathologists with senior professional titles to perform pathological diagnosis on paraffin sections, and only patients with pathological results of HCC were included in this study. All patients who participated in this study provided informed consent. This study was approved by the Research Ethics Committee. Detailed clinicopathological features of 105 HCC specimens involved in this study are shown in Table 1.

**Table 1:** Relationship between ABCA8 expression and clinicopathologic features of HCC patients (*n* = 105)

Features	ABCA8 expression		*P* Value
	Low(*n* = 47)	High (*n* = 58)	
Age			0.8078
≤ 60	24	31	
> 60	23	27	
Gender			0.9969
Male	30	37	
Female	17	21	
AFP (μg/L)			0.2034
≤ 20	11	8	
> 20	36	50	
HBV infection			0.6307
Yes	27	36	
No	20	22	
Tumor diameter (cm)			**0.0121**
≤ 5	21	40	
> 5	26	18	
metastasis			**0.0103**
Yes	28	20	
No	19	38	
TNM stage			**0.0106**
I-II	12	29	
III-IV	35	29	

### HCC cells

Human HCC cell lines, Huh7, HepG2, HCCLM3, and SK-Hep-1were obtained from the Chinese Academy of Science (Shanghai, China). Normal liver cell line WRL-68 was obtained from AcceGen (Fairfield, USA). All cell lines were cultured in Dulbecco’s Modified Eagle Medium (Gibco, USA) supplemented with 10% fetal bovine serum (Gibco, USA), 100 U/mL penicillin and 100 μg/mL streptomycin. All cells were incubated in incubators containing 5% CO_2_ at 37 °C.

### Lentivirus

Lentiviral vectors for ABCA8 and miR-374b-5p gene up-regulation (Lv-ABCA8, Lv-miR-374b-5p), down-regulation (Lv-shABCA8, Lv-anti-miR-374b-5p), empty vectors (Lv-NC) and encoding human firefly luciferase were manufactured and obtained from GeneChem (Shanghai, China). Details of the short hairpin RNA sequence against ABCA8 are listed in Additional file 1: Table S1.

### Immunoblotting analysis

Briefly, the protein samples extracted from cells or tissues were loaded, separated and then transferred onto nitrocellulose membranes (Invitrogen, Carlsbad, USA). Subsequently, 5% bovine serum albumin was used to block the nitrocellulose membrane for 1 h. Finally, the primary antibody and conjugated secondary antibody were added. Protein blots were detected using enhanced chemiluminescence (Beyotime, Shanghai, China). Details of the antibodies are listed in Additional file 1: Table S2.

### Quantitative real-time polymerase chain reaction (qPCR)

Total RNA was isolated from fresh frozen tissue and logarithmically growing cells using an RNA Miniprep Kit (Axygen, Jiangsu, China), and cDNA was synthesized using either a High Capacity RT Kit or TaqMan® MicroRNA RT kit (Applied Biosystems, Carlsbad, USA). qPCR was performed using SYBR Green (Roche, Indianapolis, USA) or TaqMan® qPCR Master Mix (Applied Biosystems, Carlsbad, USA) on a 7500 Fast PCR System. Then the expression levels of mRNA and miRNA were normalized to GAPDH and U6, respectively. Details of the primers and probes for qPCR are listed in Additional file 1: Table S3.

### Immunohistochemical (IHC) staining

After a series of processes including dewaxing, rehydration, and antigen repair, the carcinoma and adjacent non-tumor sections were blocked with secondary antibody source serum. After blocking, the sections were incubated with primary antibodies overnight. The following day, the sections were incubated with secondary antibodies and stained with diaminobenzidine. The protein staining intensity score was calculated according to previously described methods [10]. ABCA8 staining intensity was scored as 0 (negative), 1 (weak), 2 (moderate) and 3 (strong). The staining extent was scored based on the percentage of positive cells using the following scale: 0 (negative), 1 (0.01–25%), 2 (25.01–50%), 3 (50.01–75%), and 4 (75.01–100%). The histologic score (H score) for each section was calculated with the following formula: histologic score = proportion score × intensity score. Thus, the total score could be 0, 1, 2, 3, 4, 6, 8, 9, or 12, and the staining could be classified as negative/low (0, 1, 2, 3, 4) or positive/high (6, 8, 9, 12).

### Cell counting Kit-8 (CCK-8) experiments

Stably transfected cells were inoculated in 96-well plates and cultured overnight for attachment. The following day, the culture solution was replaced with a solution which contained the CCK-8 reagent and cultured for 2 h in the dark. Then, the absorbance at 450 nm was measured for each well.

### Colony formation experiments

Stably transfected cells in the logarithmic growth phase were seeded in 6-well plates for two weeks. The colonies were then fixed and stained for easy observation, and photographs were taken.

### Wound-healing assay

Stably transfected cells were inoculated in 6-well plates and cultured until cell fusion occurred. A straight cut was made at the bottom of the plate with a 10-μL pipette tip. The floating cells were washed away and the wound closure was photographed at 0 and 24 h.

### Transwell migration and invasion assay

Matrigel-coated (BD Biosciences, Franklin Lakes, NJ) or non-Matrigel-coated Transwells were used to examine the invasion and migration ability of cells. Stably transfected cells were inoculated in the upper chamber of the transwells and serum-free media was added. Normal media was injected into the plate wells. After a 24 h incubation period, the cells in the upper layer filter were removed and the cells in the bottom layer were fixed, stained and counted.

### Luciferase reporter assay

Cells were inoculated in 24-well plates, and the wild-type or mutated 3′-UTR sequence of ABCA8 were cotransfected with pRL-TK Renilla. After incubation for 48 h, luciferase activities were measured by the dual luciferase reporter assay kit.

### Immunofluorescence (IF) assay

Stably transfected cells were inoculated on glass sheets and incubated overnight. After attachment, the cells were fixed, permeabilized, and blocked with normal goat serum and incubated with primary antibodies overnight at 4 °C. The following day, cells were incubated with a fluorescent secondary antibody for 1 h. Finally, nuclei were counterstained with DAPI, and the images were photographed under a fluorescence microscope.

### Animal model

Male BALB/c nude mice (4–6 weeks old) were purchased from the Experimental Animal Center of Shanghai Institute. Subcutaneous xenograft tumors were established as follows: 5 × 10^6^ cells were dissolved in 0.15 mL phosphate buffered saline, then subcutaneously injected into the flanks of the mice. The size of the hypodermic ectopic neoplasms was observed weekly. After 6 weeks the nude mice were sacrificed and the xenograft tumor was excised. The volume of the tumor was measured by the following calculation: V=W × L × H/2.

The excised subcutaneous xenograft tumor was divided into 1 mm^3^ cubes and transplanted into the livers (left lobes) of mice from the same line to establish the orthotopic xenograft nude mice model. The mice were sacrificed after 6 weeks, after which the tumors were resected.

The pulmonary metastases nude mice model was established as follows: 4 × 10^6^ cells dissolved in 0.15 mL phosphate buffered saline were injected into the tail veins of each mouse. After 6 weeks, the mice were sacrificed and their lungs were collected. Animal experiments were approved by the Animal Ethics Committee of Harbin Medical University, and each step was carried out in accordance with animal care and use standards.

## Results

### ABCA8 is poorly expressed in HCC, and predicts poor prognosis

**Fig. 1:**
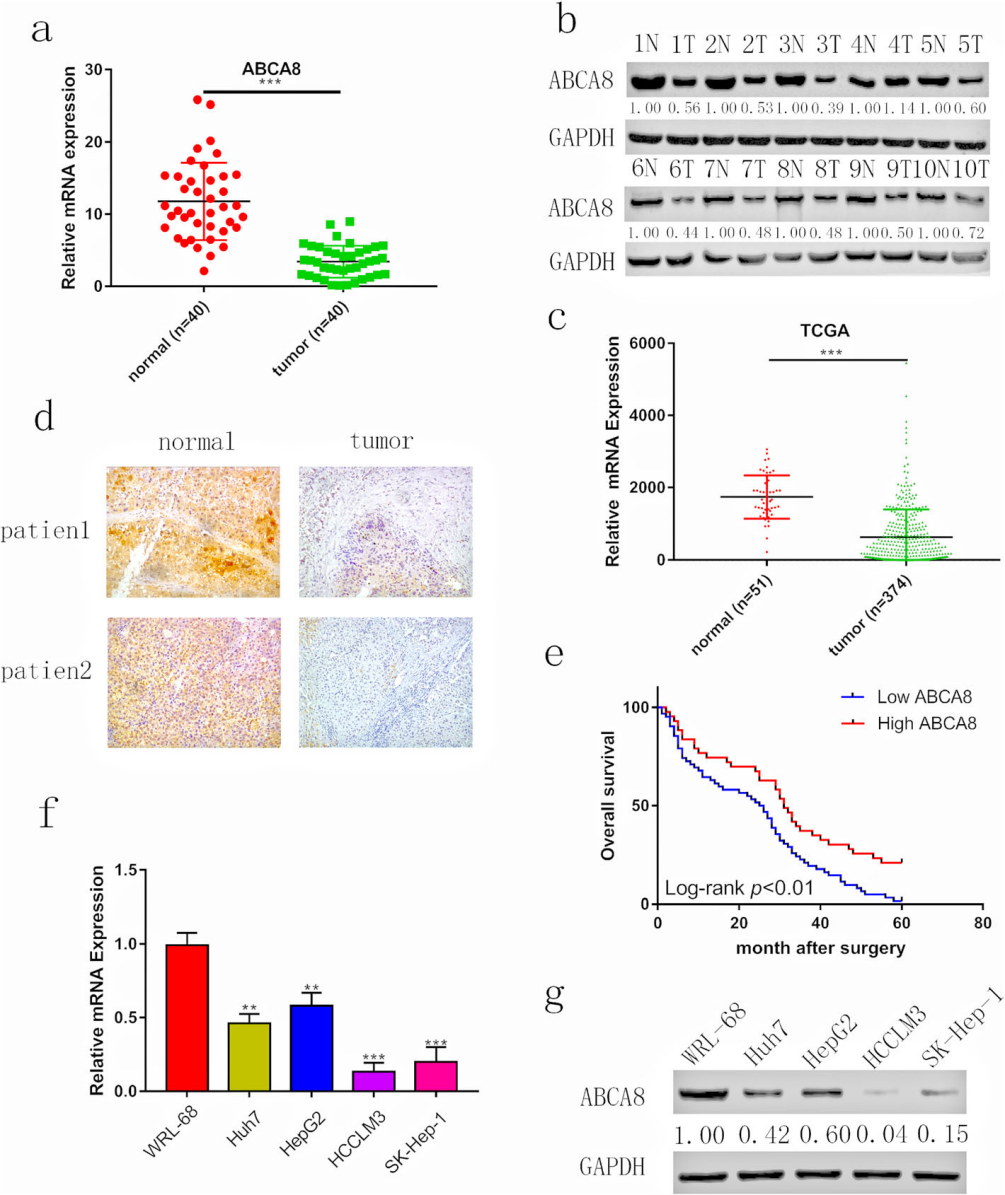
ABCA8 is reduced in human HCC and predicted poor prognosis. **a** mRNA levels of ABCA8 were analyzed in 40 HCC tissue samples and compared with adjacent non-tumor samples. **b** Protein levels of ABCA8 were analyzed in HCC tissue samples and adjacent non-tumor samples. **c** Relative ABCA8 expression levels in 374 HCC and 51 normal samples from The Cancer Genome Atlas database. **d** Representative images of ABCA8 expression detected by immunohistochemistry, Scale bars: 100× = 100 μm. **e** A Kaplan-Meier analysis of overall survival (OS) in patients with different staining of ABCA8. **f** and **g** Relative ABCA8 levels in WRL-68 and four HCC cells were analyzed using qPCR and western blotting. Data are means ± SD of three independent experiments. *p < 0.05, **p < 0.01, ***p < 0.001. T: tumor; N: adjacent non-tumor

To verify the link between ABCA8 expression and HCC, qPCR and western blots were performed. The results of these assays (Fig. 1a and b) were mostly consistent with those in the TCGA database (https://www.cancer.gov/; Fig. 1c). Additionally, immunohistochemical staining of 105 pairs of hepatocellular carcinoma and adjacent non-tumor tissue was performed (Fig. 1d) and analyzed in combination with clinicopathological characteristics. Decreased ABCA8 expression in HCC was positively correlated to tumor diameter (*p* = 0.0121), metastasis (*p* = 0.0103) and TNM stage (*p* = 0.0106) (Table 1). We also found that patients with low ABCA8 expression had poorer overall survival than patients with high ABCA8 expression (Fig. 1e), which was supported by data from UALCAN (http://ualcan.path.uab.edu/index.html; Additional file 2: Figure S1a). To confirm the reliability of our results, we consulted a Kaplan-Meier plotter (http://kmplot.com/analysis/; Additional file 2: Figure S1b), which also demonstrated that low ABCA8 expression in patients with HCC had poor prognoses. In addition, the outcomes of qPCR and western blots demonstrated that the expression of ABCA8 was lower in HCC cell lines than in normal liver cells and that the expression of ABCA8 decreased as the malignancy of the cells increased (Fig. 1f, g). These results demonstrated that ABCA8 is frequently reduced in HCC, and low expression of ABCA8 linked to poor prognosis.

### ABCA8 inhibits the growth of HCC

**Fig. 2:**
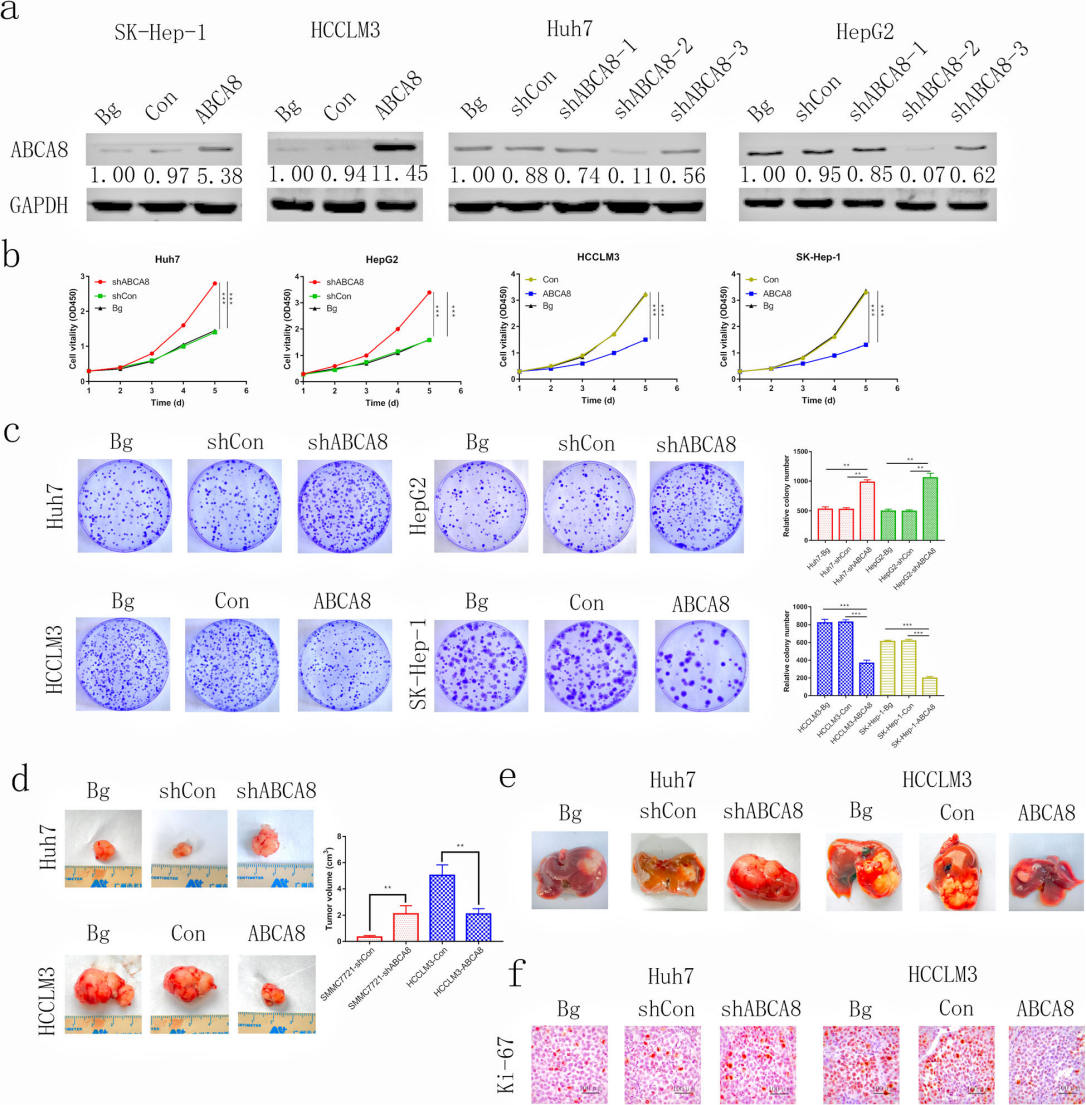
ABCA8 suppresses HCC cell proliferation and tumorigenesis in vitro and in vivo. **a** Western blot analysis of the transfection efficiency of ABCA8 in cell lines. **b** Proliferation rate was analyzed by a CCK-8 assay of indicated HCC cells. **c** Representative images of colony formation assays are shown on the left; the number of foci was counted as shown on the right. **d** and **e** Silencing expression of ABCA8 decreased Huh7 cell subcutaneous and orthotopic xenograft growth in nude mice, whereas ABCA8 overexpression had the opposite effect. Tumor volume and weight are shown on the right (*n* = 6/group). **f** Immunohistochemical detection of Ki-67 protein levels in xenograft tissues, Scale bars: 100× = 100 μm. Data are means ± SD of three independent experiments. *p < 0.05, **p < 0.01, ***p < 0.001

To understand the effect of ABCA8 in HCC, we transfected Huh7 and HepG2 cell lines with short hairpin RNAs (shRNA) by lentivirus vectors to silence the expression of ABCA8. In addition, we transfected HCCLM3 and Sk-Hep-1 cell lines with lentivirus to overexpress ABCA8. The results of transfection showed that shABCA8–2 had the most significant silencing effect (Fig. 2a and Additional file 3: Figure S2), and therefore we utilized shABCA8–2 for subsequent experiments. Meanwhile, the untreated cells were uesd as blank groups (Bg).

CCK8 experimental results revealed that overexpression of ABCA8 can inhibit the activity of HCCLM3 cells and reduce their ability to proliferate. This result was also observed in Sk-Hep-1 cells. Silencing the expression of ABCA8 can promote the vitality of Huh7 and HepG2 cells and enhance their proliferation ability (Fig. 2b). Colony formation also demonstrated that up-regulating expression of ABCA8 can reduce the number of clones formed by HCCLM3 and Sk-Hep-1 cell lines, while down-regulating expression of ABCA8 can increase the number of clones of Huh7 and HepG2 cell lines (Fig. 2c).

To further prove that ABCA8 inhibits the growth of HCC in vivo, we constructed subcutaneous and orthotopic xenograft models. Larger HCC tumors were observed in the Huh7-shABCA8 group and smaller HCC tumors were observed in the HCCLM3-ABCA8 group when compared to the corresponding control groups and black groups (Fig. 2d, e). Subcutaneous tumors were sectioned and immunohistochemical analysis showed that higher levels of Ki-67 were observed in the Huh7-shABCA8 group and lower levels of Ki-67 were observed in the HCCLM3-ABCA8 group when compared to the corresponding control groups and black groups (Fig. 2f). In short, our results showed that ABCA8 has a tumor inhibition effect both in vitro and in vivo.

### ABCA8 prohibits HCC metastasis

**Fig. 3:**
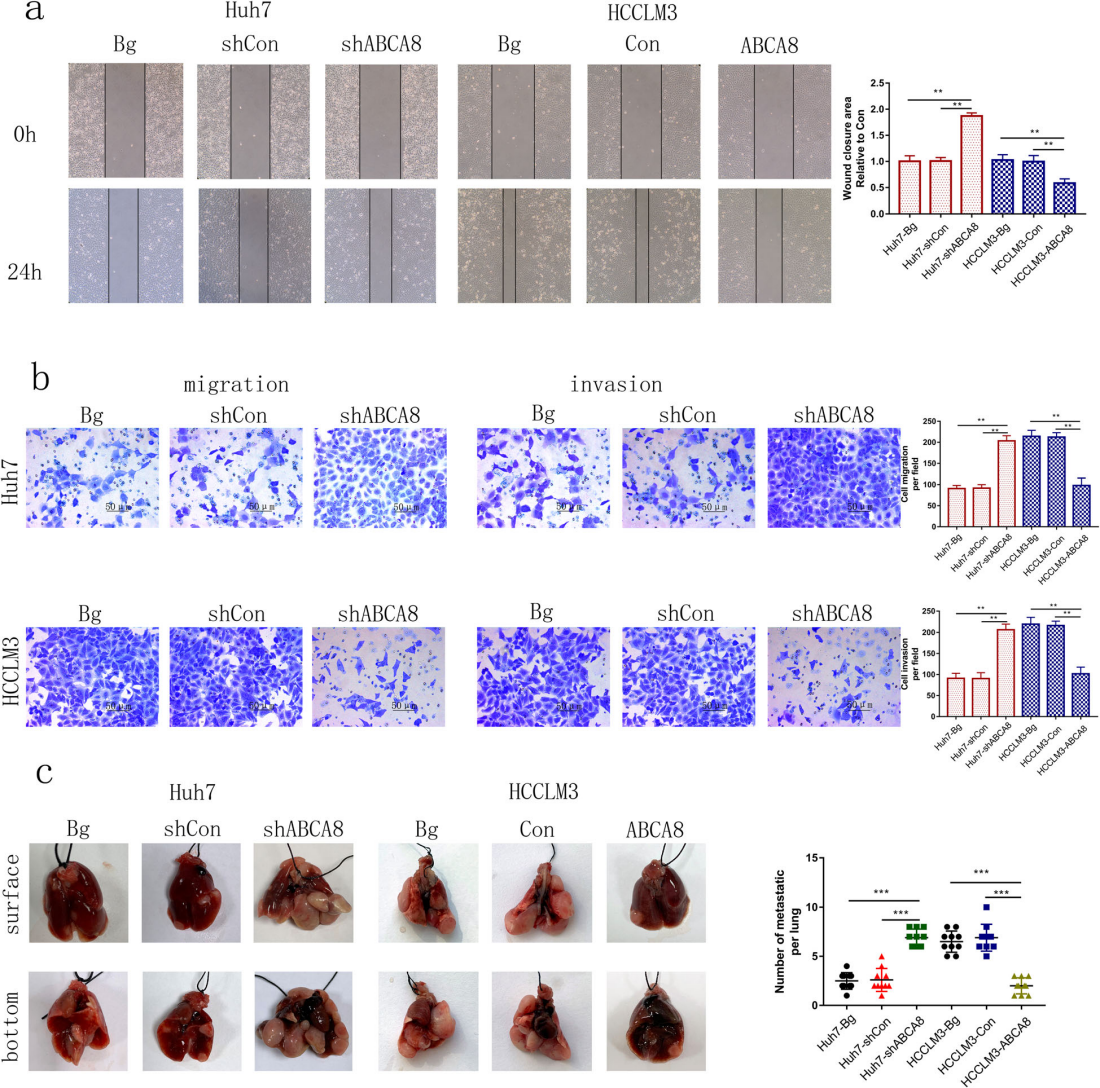
ABCA8 inhibits HCC cell migration and invasion in vitro and in vivo. **a** Representative images of the wound-healing assay **b** Transwell migration and invasion assays for indicated cell lines are shown on the left, Scale bars: 200× = 50 μm; counts of migrated and invaded HCC cells are shown on the right. **c** Representative photographs of lung tumors from indicated groups are shown on the left; the number of lung metastatic nodules in the indicated groups are shown on the right. Data are means ± SD of three independent experiments. *p < 0.05, **p < 0.01, ***p < 0.001

Metastasis is the major cause of poor prognoses for HCC patients. Therefore, we explored the effect of ABCA8 on the invasive and migratory ability of HCC cells. A wound-healing experiment showed that silencing ABCA8 accelerated the area of scratch growth in HCC cells, whereas, overexpression of ABCA8 slowed the growth of scratches (Fig. 3a and Additional file 4: Figure S3a). A transwell assay (Matrigel-coated or non-Matrigel-coated) showed that the invasive and migratory abilities of HCC cells were increased by ABCA8 silencing, whereas ABCA8 overexpression weakened the ability of cells to migrate and invade (Fig. 3b and Additional file 4: Figure S3b).

We constructed a pulmonary metastasis model (as described in our methods) to demonstrate this characteristic in vivo. We found that the Huh7-shABCA8 group had more numerous and larger pulmonary metastatic nodules than the control group. In contrast, the size and number of pulmonary metastatic nodules were suppressed in the HCCLM3-ABCA8 group compared to the control group (Fig. 3c).

### ABCA8 induces EMT via ERK signal pathway

**Fig. 4:**
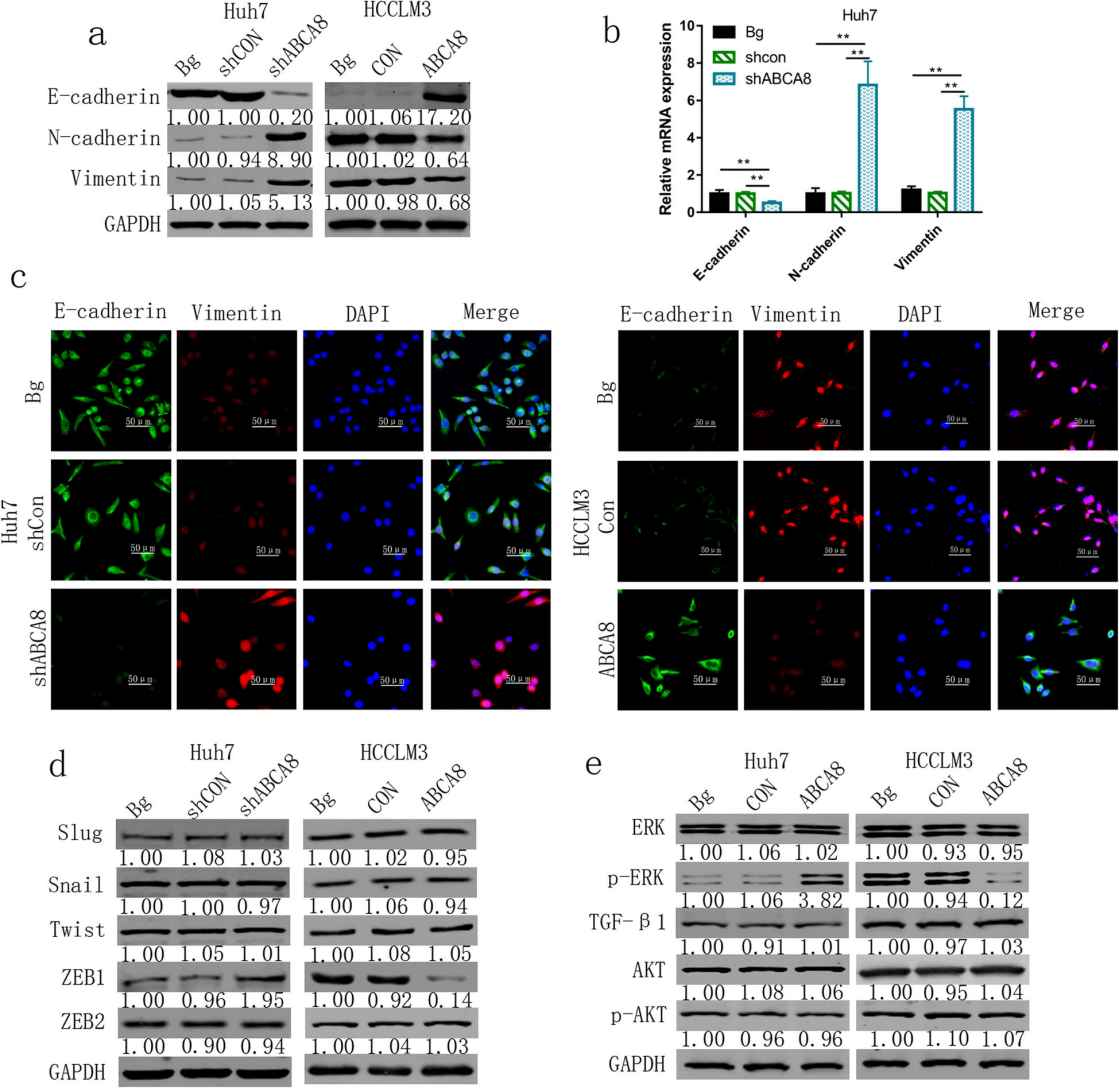
ABCA8 blocks EMT in HCC via modulating the ERK/ZEB1 axis. **a** and **b** Protein levels and mRNA levels of EMT markers measured by western blot and qPCR. **c** Representative immunofluorescence images of E-cadherin and vimentin expression in indicated HCC cell lines, Scale bars: 200× = 50 μm. **d** The expression of Slug, Snail, Twist, ZEB1, and ZEB2 were measured by western blot after ABCA8 overexpression or silencing. **e** Protein levels of ERK, p-ERK, TGF-β, and p-AKT were analyzed by western blot after ABCA8 overexpression or silencing. Data are means ± SD of three independent experiments. *p < 0.05, **p < 0.01, ***p < 0.001

Epithelial to mesenchymal transformation (EMT) is the first stage in the migration of individual and collective cells. It was thought to involve cells from the static outer membrane losing their apical polarity and cellular adhesion characteristics, eventually becoming single cells and migrating between tissues with mesenchymal phenotypes [11]. To clarify the relationship between ABCA8 and EMT, markers of EMT were evaluated by qPCR and western blots. When ABCA8 was silenced in Huh7 cells, the expression of E-cadherin (an epithelial marker) was decreased and the expression of N-cadherin and vimentin (mesenchymal markers) were increased (Fig. 4a, b and Additional file 5: Figure S4). Inversely, ABCA8 overexpression enhanced the expression of E-cadherin and decreased that of N-cadherin and vimentin in HCCLM3 cells compared to control cells. The results of immunofluorescence experiments corroborated that the reduction of ABCA8 can induce the EMT process (Fig. 4c). As we know, Snail, Slug, ZEB1/2, and Twist are important upstream transcription regulators of EMT [12–14]. However, only ZEB1 expression decreased or increased with overexpression or silencing of ABCA8, respectively (Fig. 4d). This result was confirmed by qPCR (Additional file 6: Figure S5). To clarify the molecular mechanism by which ABCA8 regulates EMT, we sought to detect key molecules in several pathways. We found that only the expression of p-ERK was up- and down-regulated by ABCA8 silencing and overexpression, respectively. Changes in p-AKT and TGF-β1 were not observed (Fig. 4e). In order to detect the expression of ERK, p-ERK, ZEB1 and EMT-related markers in vivo models, proteins were extracted from subcutaneous tumors for Western blots. The results showed that the expression of ERK and other proteins in subcutaneous tumors was basically consistent with that in cells (Additional file 7: Figure S6).

### ERK phosphorylation is critical for ABCA8-induced HCC progression

**Fig. 5:**
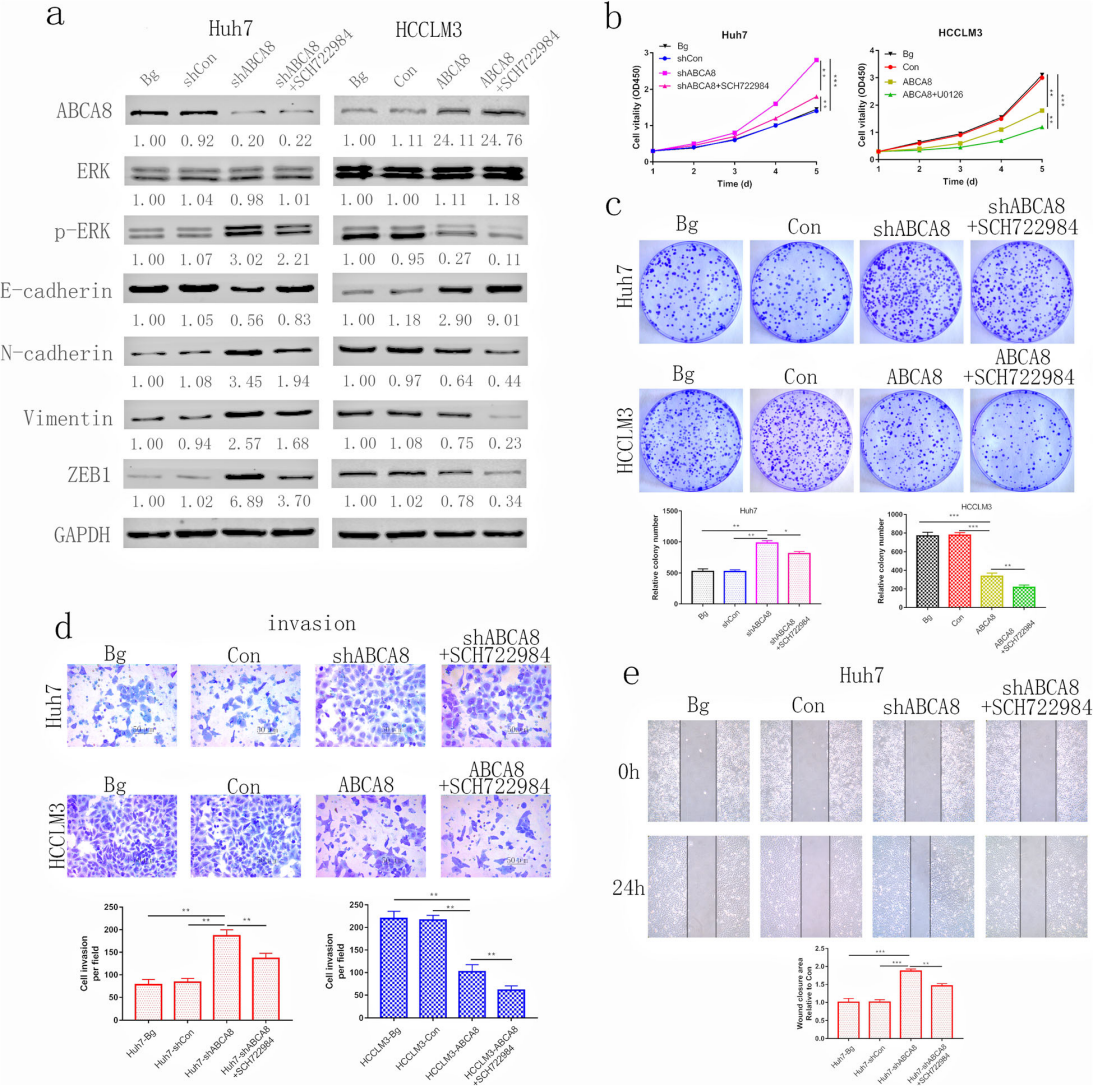
ERK phosphorylation is critical for ABCA8-induced HCC progression. **a** Western blot analysis of ABCA8, ERK, p-ERK, EMT markers and ZEB1 in indicated cells. **b** Proliferation rate after SCH722984 treatment was analyzed by CCK-8 assay of indicated HCC cells. **c** Representative images of colony formation assays are shown on the top; the number of foci counted are shown on the bottom. **d** Transwell invasion assays for indicated cell lines are shown on top, Scale bars: 200× = 50 μm; counts of invaded HCC cells are shown on the bottom. ) Wound-healing assay for indicated cell lines. Data are means ± SD of three independent experiments. *p < 0.05, **p < 0.01, ***p < 0.001

SCH722984, the novel specific inhibitor of ERK1/2 [15, 16], was used to further investigate the mechanisms of ABCA8-induced HCC progression. Western blots demonstrated that the expression of p-ERK was partially reduced by treating Huh7-shABCA8 cells with SCH722984 (Fig. 5a). An analogous effect was detected in HCCLM3-ABCA8 cells (Fig. 5a). The CCK8 assay showed that cell viability was dramatically decreased when treated with SCH722984 (Fig. 5b). The same result was observed in colony formation experiments (Fig. 5c). Similarly, when treated with SCH722984, the invasion and migration capacity of Huh7-shABCA8 and HCCLM3-ABCA8 cells was partially blocked (Fig. 5d, e and Additional file 8: Figure S7a, b). These outcomes suggest that the ERK signaling pathway is vital for ABCA8-induced HCC progression and that ERK is a key molecule in ABCA8-induced EMT.

### MiR-374b-5p directly targets ABCA8 and is correlated to poor prognosis

**Fig. 6:**
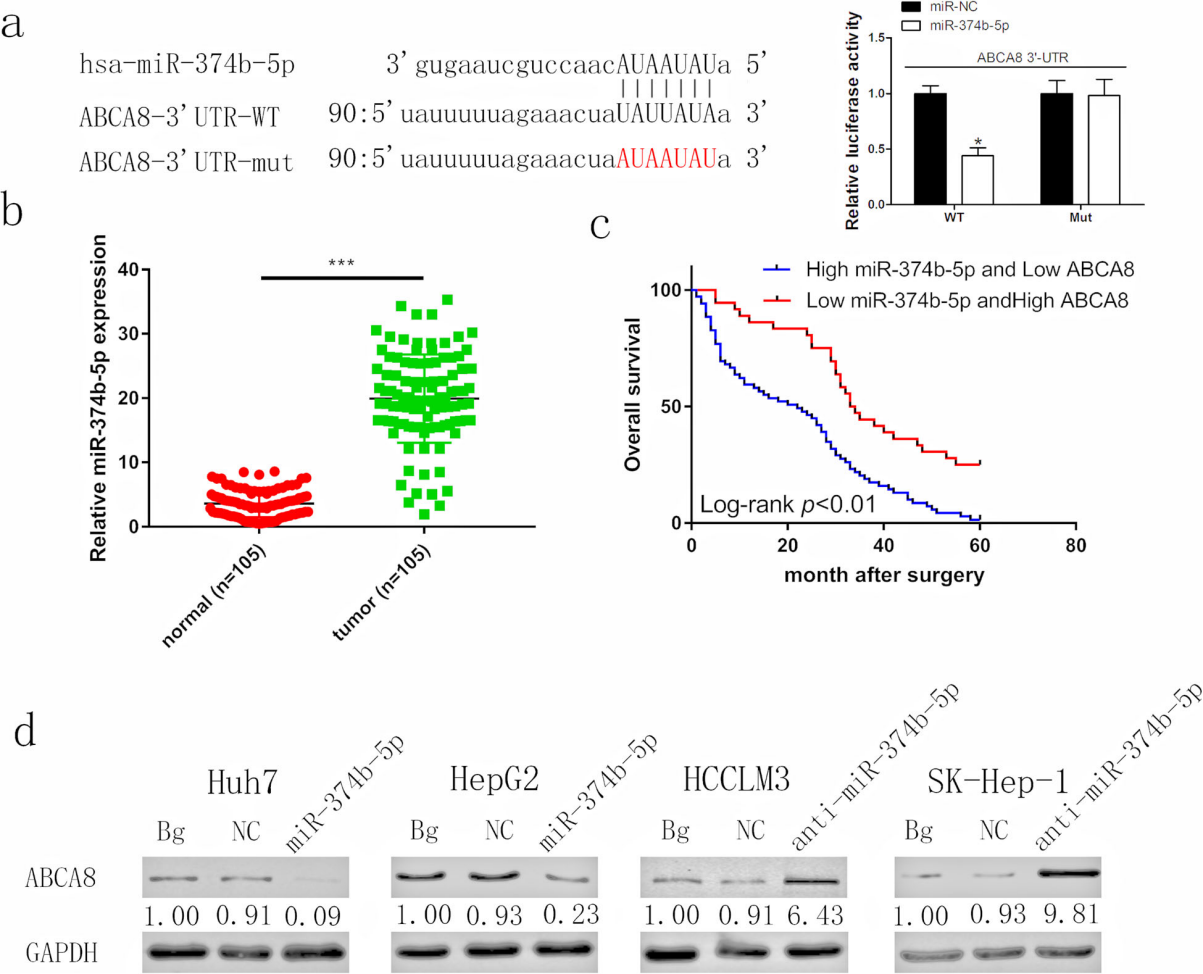
MiR-374b-5p directly targets ABCA8 and is correlated with poor prognosis. **a** Binding site of miR-374b-5p in wild-type (WT) 3′-UTR of ABCA8 and corresponding mutant type were constructed as shown on the left. The Luciferase reporter assay showed luciferase activity of HEK293T transfected with WT 3′-UTR was inhibited by miR-374b-5p overexpression. **b** Human HCC tissues displayed significantly higher miR-374b-5p levels than adjacent non-tumor tissues 105 patients. **c** Kaplan-Meier analysis indicated that the combination of low ABCA8 and high miR-374-5p predicts a poorer overall survival rate than high ABCA8 and low miR-374b-5p. **d** MiR-374-5p silencing increased ABCA8 protein levels and miR-374-5p overexpression decreased ABCA8 in indicated cells. Data are means ± SD of three independent experiments. *p < 0.05, **p < 0.01, ***p < 0.001

Many studies have shown that miRNAs can bind to the 3’UTR of mRNA, affecting its stability and function. Moreover, miRNAs are associated with many biological processes, including tumor progression [17–19]. Therefore, in order to further understand the regulatory mechanism of ABCA8 as it relates to HCC, we identified miRNAs that could be upstream regulators of ABCA8 through the publicly accessible databases, TargetScan (http://www.targetscan.org/vert_72/), miRDB (http://mirdb.org/) and miTarBase (http://mirtarbase.mbc.nctu.edu.tw/php/index.php). We screened six candidate miRNAs with highly conserved binding sequences to ABCA8 (Additional file 9: Figure S8). The miRNA inhibitors were transfected into HCC cell lines and the mRNA expression of ABCA8 was detected by qPCR (Additional file 10: Figure S9). Among the six miRNAs tested, ABCA8 levels only increased when miR-374b-5p was inhibited. Previous studies have reported that miR-374b-5p is highly expressed in HCC and that high levels of miR-374b-5p expression can promote tumor proliferation and metastasis [20]. This result in combination with our own data led us to focus our subsequent experiments on miR-374b-5p. Through a luciferase reporter assay, we tested for a link between miR-374b-5p and ABCA8. As expected, luciferase activity was suppressed by miR-374b-5p in the wild-type (WT) ABCA8 3′-UTR group, but no change was detected for the mutant group (Fig. 6a). qPCR illustrated that miR-374b-5p was upregulated in HCC tissues (Fig. 6b) and HCC cell lines (Additional file 11: Figure S10). Our results are consistent with previous reports [20]. Furthermore, clinicopathological analysis showed that high levels of miR-374b-5p expression were positively correlated with tumor diameter (*p* = 0.0166), metastasis (*p* = 0.0021) and TNM stage (*p* = 0.0090, Table 2). Patients with high levels of miR-374b-5p and low levels of ABCA8 had lower survival rates compared with patients with low levels of miR-374b-5p and high levels of ABCA8 (Fig. 6c). We transfected Huh7 and HepG2 cell lines with lentivirus-miR-374b-5p and HCCLM3 and SK-Hep-1 cell lines with lentivirus-anti-miR-374b-5p, followed by western blotting to verify changes in ABCA8 expression levels (Fig. 6d). These results demonstrated that miR-374b-5p directly targets ABCA8 in HCC. The high expression of miR-374b-5p combined with low expression of ABCA8 was correlated to a poor prognosis for HCC patients.

**Table 2:** Relationship between miR-374b-5p expression and clinicopathologic features of HCC patients (*n* = 105)

Features	miR-374b-5p expression		*P* Value
	Low(*n* = 55)	High (*n* = 50)	
Age			0.3914
≤ 60	31	24	
> 60	24	26	
Gender			0.4386
Male	37	30	
Female	18	20	
AFP (μg/L)			0.3217
≤ 20	8	11	
> 20	47	39	
HBV infection			0.6900
Yes	34	29	
No	21	21	
Tumor diameter (cm)			**0.0166**
≤ 5	38	23	
> 5	17	27	
metastasis			**0.0021**
Yes	33	15	
No	22	35	
TNM stage			**0.0090**
I-II	28	13	
III-IV	27	37	

### MiR-374b-5p promotes HCC progression via the ABCA8/ERK/ZEB1 axis

**Fig. 7:**
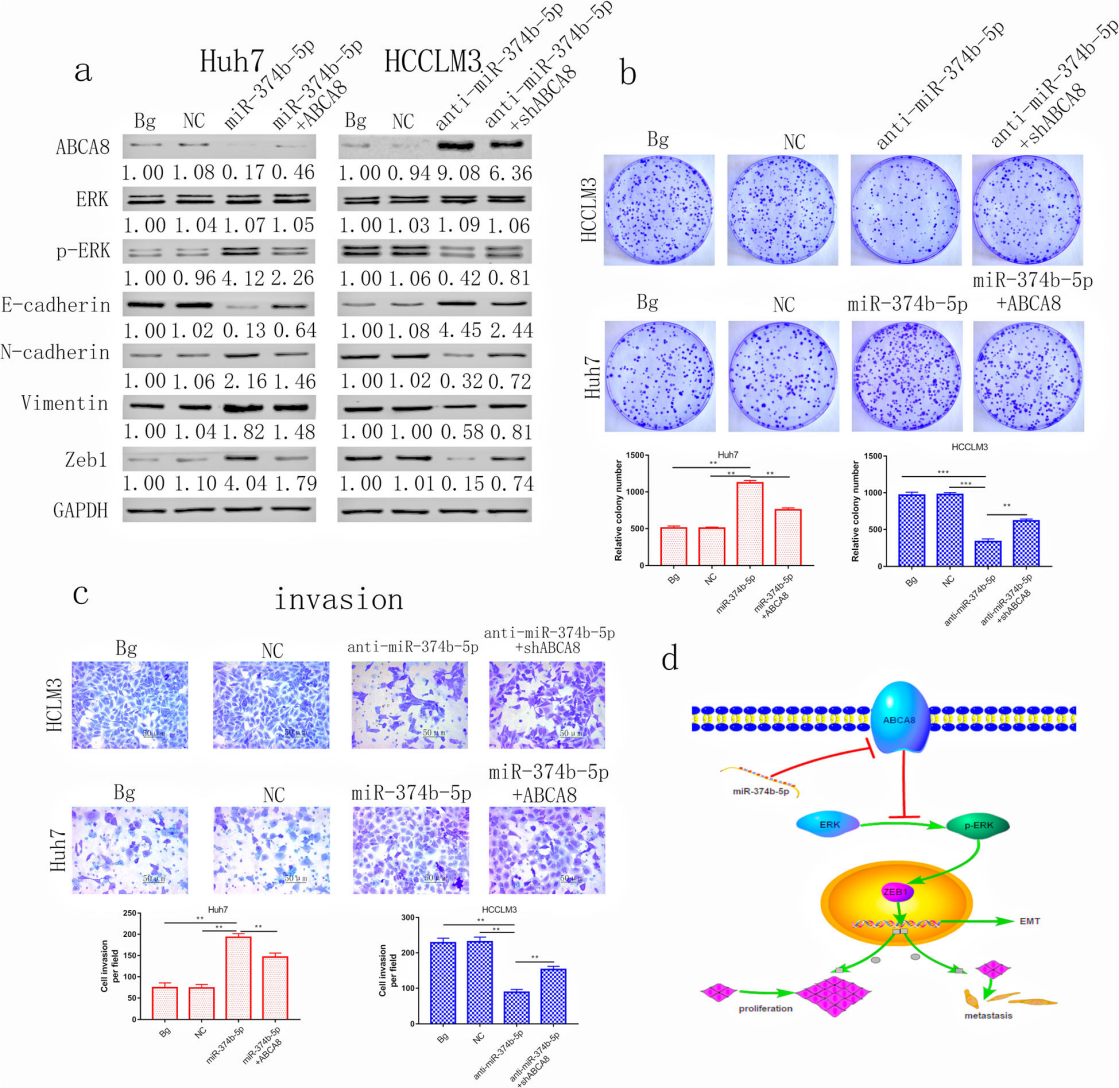
ABCA8 is regulated by miR-374b-5p and inhibits the progression of HCC via the ERK/ZEB1 axis. **a** Western blot analysis of the effects of miR-374b-5p and ABCA8 on ERK/ZEB1 axis induced EMT. Overexpression of ABCA8 in Huh7-mir-374b-5p cells can weaken the phosphorylation of ERK and inhibit EMT. In contrast, silencing ABCA8 in HCCLM3-anti-mir-374b-5p cells enhance the phosphorylation of ERK and induce EMT. **b** Representative images of colony formation assays for indicated cells. **c** Transwell invasion assays for indicated cell lines are shown on top, Scale bars: 200× = 50 μm; counts of invaded HCC cells are shown on the bottom. **d** Schematic representation of the mechanism underlying ABCA8-mediated HCC progression. Data are means ± SD of three independent experiments. *p < 0.05, **p < 0.01, ***p < 0.001

The outcome of western blots demonstrated that the expression of ABCA8 and E-cadherin were up-regulated after miR-374b-5p silencing, while the expression of p-ERK, N-cadherin, Vimentin and ZEB1 were down-regulated. ABCA8 silencing can partially restore this effect, while the expression of total ERK was not affected by either miR-374b-5p silencing or ABCA8 silencing in HCCLM3 cell line (Fig. 7a). Overexpression of miR-374b-5p in the Huh7 cell line had the opposite effect and these effects could be reversed by overexpression of ABCA8.

In order to detect the role of miR-374b-5p on cell proliferation, colony formation experiments were performed. Overexpression of miR-374b-5p promoted HCC cell proliferation, which could be partially blocked by overexpression of ABCA8. Silencing miR-374b-5p had the opposite effect and that effect is partially reversed by silencing ABCA8 (Fig. 7b). Transwell assays (migration or invasion) indicated that silencing miR-374b-5p inhibited the migratory and invasive capability of HCCLM3 cells, and the overexpression of miR374b-5p facilitated Huh7 cell migration and invasion. The migratory and invasive capabilities, that are strengthened or weakened by overexpression or silencing of miR-374b-5p, can be partially reversed by overexpression or silencing of ABCA8, respectively (Fig. 7c and Additional file 12: Figure S11). These outcomes illustrate that miR-374b-5p promotes the development of HCC through the ABCA8/ERK/Zeb1 axis (Fig. 7d).

## Discussion

HCC is a disease of great concern due to its high malignancy and insensitivity to radiotherapy and chemotherapy [21]. Although surgical treatments such as hepatectomy and liver transplantation can delay the progression of HCC to some extent, the 5-year survival of patients is not ideal [22, 23]. Therefore, it is of great importance to find an effective target for the treatment of HCC. Our study is the first to elucidate the role of ABCA8 in cancer, particularly in HCC.

We revealed the role of ABCA8 in HCC progression. Our evidence shows that the expression of ABCA8 is significantly decreased in HCC tissues and HCC cell lines when compared to adjacent non-tumor tissues and normal liver cells. Low expression of ABCA8 was associated with increased tumor size, metastasis, and a more advanced TNM stage. Patients with low levels of ABCA8 had worse prognoses than those with high levels of ABCA8. Consistent with the characteristics of clinical cases, we found that ABCA8 can inhibit the proliferation, invasion, and migration of tumor cells in vivo and in vitro.

EMT is the initial step in inducing tumor cell metastasis [11]. EMT participates in a variety of biological and pathological processes, such as embryo formation, tissue regeneration, and tumorigenesis [24–26]. Accumulating evidence has demonstrated that EMT acts in a critical role during the metastasis of many types of tumors, including HCC [27, 28]. However, some aspects of EMT remain unclear and further research is needed to relate the clinical management of HCC with EMT-related biomarkers and targeted therapy [27]. Importantly, we found that dysregulated ABCA8 can alter epithelial and mesenchymal markers and promote EMT. Among several transcription factors that regulate EMT, only levels of ZEB1 were effected by ABCA8 levels. This is the first time the potential mechanism (inducing EMT in HCC) of ABCA8 in cancer has been revealed. A large body of evidence indicates that many signaling pathways are over-activated or deactivated in the induction of EMT and the promotion of carcinogenesis [29–31]. We examined key pathways and found that when ABCA8 was overexpressed or silenced, only the protein content of phosphorylated ERK was significantly changed, while total ERK was not changed. The ERK pathway is well studied, and a large number of experiments have confirmed its association with EMT [32–34]. After transfected ABCA8 cells were treated with SCH722984, ABCA8-induced EMT and tumorigenesis was partially blocked. This suggests that the ERK pathway is crucial for ABCA8-regulated HCC processes.

However, in our study, ABCA8 was found to inhibit the EMT process in HCC by inhibiting the activation of ERK, the specific activation mode is still unclear. It has been previously reported that ABCA8 is a transmembrane transporter responsible for regulates cholesterol efflux and HDL cholesterol levels [35]. Therefore, the low expression of ABCA8 inevitably blocks the outflow of cholesterol, allowing cholesterol to accumulate in cells. Previous studies have shown that excess intracellular cholesterol can induce oxidative stress and then generate Reactive oxygen species (ROS) [36, 37]. ERK signaling pathway will be activated as ROS increases, which finally promote the growth and metastasis of pancreatic cancer [38], therefore we put more attention on the ERK signaling pathway. It has been indicated that ERK signaling pathway can not only promote cell proliferation, apoptosis and metastasis but also induce EMT [39–41]. Therefore, we speculated that ABCA8 induces oxidative stress and ROS production through intracellular accumulation of cholesterol, which then induces EMT through the activation of ERK signaling pathway and facilitates the growth and metastasis of HCC cells. Liver is the most important organ for the synthesis of cholesterol in human body. The specific amount of cholesterol in the liver is increased due to the reduction of ABCA8, and the exact pathway of ROS production, cellular oxidative stress and other details after cholesterol increase need to be further explored in the follow-up studies.

Recent reports on miRNA reveal it has an indispensable role in tumorigenesis and development [42]. We clarified the regulatory mechanism of ABCA8 in HCC by demonstrating that miR-374b-5p directly targets ABCA8 and down-regulates the expression of ABCA8 via a luciferase reporter assay. miR-374b-5p is known to be highly expressed in HCC [20], in our study, altering the expression levels of miR-374b-5p regulated the expression of ABCA8; the proliferation, invasiveness, and migration of HCC cells; and regulated EMT through the ERK pathway. Furthermore, high expression of miR-374b-5p was associated with an increase in tumor size, metastasis, and a more advanced TNM stage.

## Conclusions

Our results indicate that ABCA8 is down-regulated in HCC tissues and cell lines. ABCA8 expression is negatively correlated with HCC progression and prognosis. Moreover, ABCA8 is the direct target of miR-374b-5p, and inhibits the ERK/ZEB1 signaling pathway. Our work is the first to elucidate the role of ABCA8 in cancer, particularly in HCC, and ABCA8 is expected to be a new therapeutic target for HCC.

## Supplementary information

**Additional file 1: Table S1.** The sequences of the Lv-shRNAs. **Table S2.** Primary antibodies for WB, IHC, and IF. **Table S3.** Sequence of Primers for qPCR.

**Additional file 2: Figure S1.** Available database information for ABCA8 survival correlation analysis. (a) Survival analysis of ABCA8 in the UALCAN database. (b) Survival analysis of ABCA8 in the Kaplan-Meier plotter database.

**Additional file 3: Figure S2.** qPCR analysis of ABCA8 expression after ABCA8 upregulation or silencing in HCC cells. Data are means ± SD of three independent experiments. *p < 0.05, **p < 0.01, ***p < 0.001.

**Additional file 4: Figure S3.** ABCA8 inhibits migration and invasion of HepG2 and SK-Hep-1 cells. (a) Representative images from the wound-healing assay using indicated cell lines. (b) Transwell migration and invasion assays for indicated cell lines are shown on the left, Scale bars: 200× = 50 μm; cell counts are shown on the right. Data are means ± SD of three independent experiments. *p < 0.05, **p < 0.01, ***p < 0.001.

**Additional file 5: Figure S4.** mRNA level of EMT markers measured by qPCR. *p < 0.05, **p < 0.01, ***p < 0.001.

**Additional file 6: Figure S5.** qPCR determined ABCA8 overexpression and silencing decreased and increased ZEB1 expression, respectively. No changes were observed in Snail, Slug, Twist and ZEB2 mRNA levels. *p < 0.05, **p < 0.01, ***p < 0.001.

**Additional file 7: Figure S6.** Western blot was used to analyze the expression of ABCA8, ERK, p-ERK and EMT-related marker proteins in subcutaneous tumors.

**Additional file 8: Figure S7.** SCH722984 partially blocks ABCA8-induced HCC migration and invasion. (a) Transwell migration assays for indicated cell lines are shown on the left, Scale bars: 200× = 50 μm; cell counts are shown on the right. (b) Representative images from the wound-healing assay using indicated cell lines. Data are means ± SD of three independent experiments. *p < 0.05, **p < 0.01, ***p < 0.001.

**Additional file 9: Figure S8.** Analysis using TargetScan, miRDB, and miRanda revealed several miRNAs that might regulate ABCA8.

**Additional file 10: Figure S9.** Real-time PCR analysis of ABCA8 levels in four HCC cell lines following transfection of miRNA inhibitors. *p < 0.05, **p < 0.01, ***p < 0.001.

**Additional file 11: Figure S10.** qPCR analysis of miR-374b-5p levels in four HCC cell lines. Data are means ± SD of three independent experiments. *p < 0.05, **p < 0.01, ***p < 0.001.

**Additional file 12: Figure S11.** Transwell migration assays for indicated cell lines are shown on top, Scale bars: 200× = 50 μm; cell counts are shown on bottom. Data are means ± SD of three independent experiments. *p < 0.05, **p < 0.01, ***p < 0.001.

## Data Availability

All data related to this study are included in this paper and its supplementary information files.

## Abbreviations

HCC: Hepatocellular carcinoma
ABCA8: ATP binding cassette subfamily A member 8
qPCR: Quantitative polymerase chain reaction
ERK: Extracellular signal-regulated kinase
ZEB1: Zinc finger E-box binding homeobox 1
UTR: Untranslated region

## References

[1] Bray F, Ferlay J, Soerjomataram I, Siegel RL, Torre LA, Jemal A (2018). Global cancer statistics 2018: GLOBOCAN estimates of incidence and mortality worldwide for 36 cancers in 185 countries. CA Cancer J Clin.

[2] Goh GB, Chang PE, Tan CK (2015). Changing epidemiology of hepatocellular carcinoma in Asia. Best Pract Res Clin Gastroenterol.

[3] Ferlay J, Colombet M, Soerjomataram I, Mathers C, Parkin DM, Piñeros M, Znaor A, Bray F (2019). Estimating the global cancer incidence and mortality in 2018: GLOBOCAN sources and methods. Int J Cancer.

[4] Singal AG, El-Serag HB (2015). Hepatocellular carcinoma from epidemiology to prevention: translating knowledge into practice. Clin Gastronenterol Hepatol.

[5] Khemlina G, Ikeda S, Kurzrock R (2017). The biology of hepatocellular carcinoma: implications for genomic and immune therapies. Mol Cancer.

[6] Annilo T, Chen ZQ, Shulenin S, Dean M (2003). Evolutionary analysis of a cluster of ATP-binding cassette (ABC) genes. Mamm Genome.

[7] Pan H, Zheng Y, Pan Q, Chen H, Chen F, Wu J, Di D (2019). Expression of LXR-β, ABCA1 and ABCG1 in human triple-negative breast cancer tissues. Oncol Rep.

[8] Sharma B, Agnihotri N (2019). Role of cholesterol homeostasis and its efflux pathways in cancer progression. J Steroid Biochem Mol Biol.

[9] D'Amore S, Härdfeldt J, Cariello M, Graziano G, Copetti M, Di Tullio G (2018). Identification of miR-9-5p as direct regulator of ABCA1 and HDL-driven reverse cholesterol transport in circulating CD14+ cells of patients with metabolic syndrome. Cardiovasc Res.

[10] Wang Y, Liang Y, Yang G, Lan Y, Han J, Wang J (2018). Tetraspanin 1 promotes epithelial-to-mesenchymal transition and metastasis of cholangiocarcinoma via PI3K/AKT signaling. J Exp Clin Cancer Res.

[11] Barriga EH, Mayor R (2019). Adjustable viscoelasticity allows for efficient collective cell migration. Semin Cell Dev Biol.

[12] Ma Y, Zhang H, Xiong C, Liu Z, Xu Q, Feng J (2018). CD146 mediates an E-cadherin-to-N-cadherin switch during TGF-β signaling-induced epithelial-mesenchymal transition. Cancer Lett.

[13] Kim TW, Lee SY, Kim M, Cheon C, Jang BH, Shin YC, Ko SG (2018). DSGOST regulates resistance via activation of autophagy in gastric cancer. Cell Death Dis.

[14] Yao L, Conforti F, Hill C, Bell J, Drawater L, Li J (2019). Paracrine signalling during ZEB1-mediated epithelial-mesenchymal transition augments local myofibroblast differentiation in lung fibrosis. Cell Death Differ.

[15] Wong DJ, Robert L, Atefi MS, Lassen A, Avarappatt G, Cerniglia M (2014). Antitumor activity of the ERK inhibitor SCH772984 [corrected] against BRAF mutant, NRAS mutant and wild-type melanoma. Mol Cancer.

[16] Morris EJ, Jha S, Restaino CR, Dayananth P, Zhu H, Cooper A (2013). Discovery of a novel ERK inhibitor with activity in models of acquired resistance to BRAF and MEK inhibitors. Cancer Discov.

[17] Liu H, Bi J, Dong W, Yang M, Shi J, Jiang N (2018). Invasion-related circular RNA circFNDC3B inhibits bladder cancer progression through the miR-1178-3p/G3BP2/SRC/FAK axis. Mol Cancer.

[18] Liang AL, Zhang TT, Zhou N, Wu CY, Lin MH, Liu YJ (2016). MiRNA-10b sponge: an anti-breast cancer study in vitro. Oncol Rep.

[19] Rokavec M, Horst D, Hermeking H (2017). Cellular model of Colon Cancer progression reveals signatures of mRNAs, miRNA, IncRNAs, and epigenetic modifications associated with metastasis. Cancer Res.

[20] Yin Z, Ma T, Yan J, Shi N, Zhang C, Lu X (2019). LncRNA MAGI2-AS3 inhibits hepatocellular carcinoma cell proliferation and migration by targeting the miR-374b-5p/SMG1 signaling pathway. Journal Cell Physiol.

[21] Kalogeridi MA, Zygogianni A, Kyrgias G, Kouvaris J, Chatziioannou S, Kelekis N, Kouloulias V (2015). Role of radiotherapy in the management of hepatocellular carcinoma: a systematic review. World J Hepatol.

[22] Yang SL, Liu LP, Sun YF, Yang XR, Fan J, Ren JW (2016). Distinguished prognosis after hepatectomy of HBV-related hepatocellular carcinoma with or without cirrhosis: a long-term follow-up analysis. J Gastroenterol.

[23] Iguchi T, Shirabe K, Aishima S, Wang H, Fujita N, Ninomiya M (2015). New pathologic stratification of microvascular invasion in hepatocellular carcinoma: predicting prognosis after living-donor liver transplantation. Transplantation..

[24] Kalluri R, Weinberg RA (2009). The basics of epithelial-mesenchymal transition. J Clin Invest.

[25] Thiery JP, Sleeman JP (2006). Complex networks orchestrate epithelial-mesenchymal transitions. Nat Rev Mol Cell Biol.

[26] Yang J, Weinberg RA (2008). Epithelial-mesenchymal transition: at the crossroads of development and tumor metastasis. Dev Cell.

[27] Giannelli G, Koudelkova P, Dituri F, Mikulits W (2016). Role of epithelial to mesenchymal transition in hepatocellular carcinoma. J Hepatol.

[28] Yoshida S, Kornek M, Ikenaga N, Schmelzle M, Masuzaki R, Csizmadia E (2013). Sublethal heat treatment promotes epithelial-mesenchymal transition and enhances the malignant potential of hepatocellular carcinoma. Hepatology.

[29] Cui Y, Sun D, Song R, Zhang S, Liu X, Wang Y (2019). Upregulation of cystatin SN promotes hepatocellular carcinoma progression and predicts a poor prognosis. J Cell Physiol.

[30] Brockhausen J, Tay SS, Grzelak CA, Bertolino P, Bowen DG, d'Avigdor WM (2015). miR-181a mediates TGF-β-induced hepatocyte EMT and is dysregulated in cirrhosis and hepatocellular cancer. Liver Int.

[31] Xie B, Lin W, Ye J, Wang X, Zhang B, Xiong S (2015). DDR2 facilitates hepatocellular carcinoma invasion and metastasis via activating ERK signaling and stabilizing SNAIL1. J Exp Clin Cancer Res.

[32] Xie L, Law BK, Chytil AM, Brown KA, Aakre ME, Moses HL (2004). Activation of the Erk pathway is required for TGF-beta1-induced EMT in vitro. Neoplasia..

[33] Buonato JM, Lazzara MJ (2014). ERK1/2 blockade prevents epithelial-mesenchymal transition in lung cancer cells and promotes their sensitivity to EGFR inhibition. Cancer Res.

[34] Grände M, Franzen A, Karlsson JO, Ericson LE, Heldin NE, Nilsson M (2002). Transforming growth factor-beta and epidermal growth factor synergistically stimulate epithelial to mesenchymal transition (EMT) through a MEK-dependent mechanism in primary cultured pig thyrocytes. J Cell Sci.

[35] Trigueros-Motos L, van Capelleveen JC, Torta F, Castaño D, Zhang LH, Chai EC (2017). ABCA8 regulates cholesterol efflux and high-density lipoprotein cholesterol levels. Arterioscler Thromb Vasc Biol.

[36] Hermida N, Balligand JL (2014). Low-density lipoprotein-cholesterol-induced endothelial dysfunction and oxidative stress: the role of statins. Antioxid Redox Signal.

[37] Seo E, Kang H, Choi H, Choi W, Jun HS (2019). Reactive oxygen species-induced changes in glucose and lipid metabolism contribute to the accumulation of cholesterol in the liver during aging. Aging Cell.

[38] Cheung EC, DeNicola GM, Nixon C, Blyth K, Labuschagne CF, Tuveson DA, Vousden KH (2020). Dynamic ROS Control by TIGAR Regulates the Initiation and Progression of Pancreatic Cancer. Cancer Cell.

[39] Sheng W, Shi X, Lin Y, Tang J, Jia C, Cao R (2020). Musashi2 promotes EGF-induced EMT in pancreatic cancer via ZEB1-ERK/MAPK signaling. J Exp Clin Cancer Res.

[40] Wang J, Zhang Z, Li R, Mao F, Sun W, Chen J (2018). ADAM12 induces EMT and promotes cell migration, invasion and proliferation in pituitary adenomas via EGFR/ERK signaling pathway. Biomed Pharmacother.

[41] Ichikawa K, Kubota Y, Nakamura T, Weng JS, Tomida T, Saito H, Takekawa M (2015). MCRIP1, an ERK substrate, mediates ERK-induced gene silencing during epithelial-mesenchymal transition by regulating the co-repressor CtBP. Mol Cell.

[42] Murakami Y, Yasuda T, Saigo K, Urashima T, Toyoda H, Okanue T, Shimotohno K (2006). Comprehensive analysis of microRNA expression patterns in hepatocellular carcinoma and non-tumorous tissues. Oncogene..

