# Supplementary material for: ABCA8 is regulated by miR-374b-5p and inhibits proliferation and metastasis of hepatocellular carcinoma through the ERK/ZEB1 pathway
Source: J Exp Clin Cancer Res. 2020 May 19;39:90. doi: 10.1186/s13046-020-01591-1 (PMC7236190; doi:10.1186/s13046-020-01591-1)
Supplement: Supplementary file 1 — Additional file 1: Table S1. The sequences of the Lv-shRNAs. Table S2. Primary antibodies for WB, IHC, and IF. Table S3. Sequence of Primers for qPCR. [file 13046_2020_1591_MOESM1_ESM.docx]

Table S1. The sequences of the Lv-shRNAs.

| shRNAs Target sequences |
| --- |
| shCon TTCTCCGAACGTGTCACGT  shABCA8-1   AGTTCTTATGGACATTGTT  shABCA8-2 AAGGCCAAATCACTGCAAT  shABCA8-3 TGGGTCATAGTATCTGATA |

Table S2. Primary antibodies for WB, IHC and IF

| Antibody | Concentration  for WB | Concentration  for IHC | Concentration  for IF | Specificity | Company |
| --- | --- | --- | --- | --- | --- |
| ABCA8 | 1:1000 | 1:100 |  | Rabbit polyclonal | Abcam |
| E-cadherin | 1:1000 |  | 1:100 | Mouse monoclonal | Abcam |
| N-cadherin | 1:1000 |  |  | Rabbit polyclonal | Abcam |
| Vimentin | 1:2000 |  | 1:200 | Rabbit polyclonal | Abcam |
| TGF-β1 | 1:1000 |  |  | Rabbit monoclonal | Abcam |
| AKT | 1:1000 |  |  | Rabbit monoclonal | CST |
| p-AKT | 1:1000 |  |  | Rabbit monoclonal | CST |
| ERK1/2 | 1:1000 |  |  | Rabbit monoclonal | CST |
| p-ERK1/2 | 1:1000 |  |  | Rabbit monoclonal | CST |
| Snail | 1:1000 |  |  | Goat polyclonal | Abcam |
| Slug | 1:1000 |  |  | Rabbit polyclonal | Abcam |
| Twist | 1:1000 |  |  | Mouse monoclonal | Abcam |
| ZEB1 | 1:1000 |  |  | Rabbit monoclonal | Abcam |
| ZEB2 | 1:1000 |  |  | Rabbit polyclonal | Abcam |
| Ki-67 |  | 1:200 |  | Rabbit polyclonal | Abcam |

Table S3. Sequence of Primers for PCR

| Primers |  | Sequences (5’------3’) |
| --- | --- | --- |
| ABCA8 | Forward： | 5’-GCTTAGTCCCTTTGCCTTCA-3’ |
|  | Reverse： | 5’-TCGCCAATGCCAGATAGAG-3’. |
| E-cadherin | Forward： | 5’-TTGCTACTGGAACAGGGACAC-3’ |
|  | Reverse： | 5’-GATGTATTGGGAGGAAGGTCTG-3’ |
| N-cadherin | Forward： | 5’-CCGACGAATGGATGAAAGAC-3’ |
|  | Reverse： | 5’-AGTCAAACACTAACAGGGAGTCA-3’ |
| Vimentin | Forward： | 5’-TTGAACGCAAAGTGGAATC-3’ |
|  | Reverse： | 5’-AGGTCAGGCTTGGAAACA-3’ |
| GAPDH | Forward： | 5’-GCACCGTCAAGGCTGAGAAC-3’ |
|  | Reverse： | 5’-TGGTGAAGACGCCAGTGGA-3’ |
| Snail | Forward： | 5’-TTTCTGGTTCTGTGTCCTCTG-3’ |
|  | Reverse： | 5’-TGTCAGCCTTTGTCCTGTAGC-3’ |
| Slug | Forward： | 5’-CCTCCATCTGACACCTCC-3’ |
|  | Reverse： | 5’-CCCAGGCTCACATATTCC-3’ |
| Twist | Forward： | 5’-CGACGACAGCCTGAGCAACA-3’ |
|  | Reverse： | 5’-CCACAGCCCGCAGACTTCTT-3’ |
| ZEB1 | Forward： | 5’-AAGTGGCGGTAGATGGTA-3’ |
|  | Reverse： | 5’-TTGTAGCGACTGGATTTT-3’ |
| ZEB2 | Forward： | 5’-ACCAGCGGAAACAAGGATTT-3’ |
|  | Reverse： | 5’-TATGTCGCAGAAGGGAACTG-3’ |
| hsa-miR-194-5p TaqMan™ MicroRNA (Life Technologies, 002355, Cat#: 4427975) | | |
