# Supplementary figures and images for: ABCA8 is regulated by miR-374b-5p and inhibits proliferation and metastasis of hepatocellular carcinoma through the ERK/ZEB1 pathway
Source: J Exp Clin Cancer Res. 2020 May 19;39:90. doi: 10.1186/s13046-020-01591-1 (PMC7236190; doi:10.1186/s13046-020-01591-1)

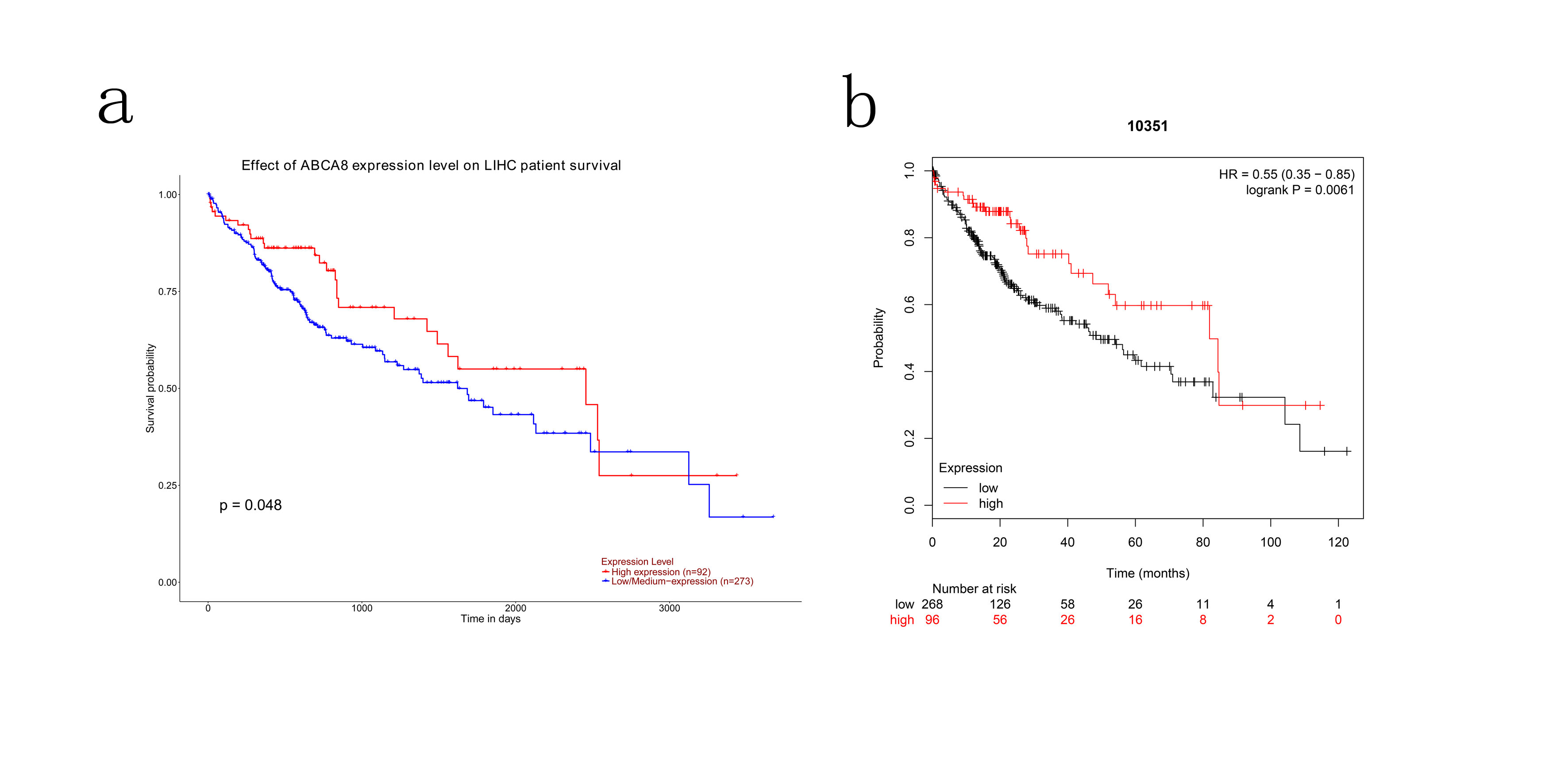

Supplement: Supplementary file 2 — Additional file 2: Figure S1. Available database information for ABCA8 survival correlation analysis. (a) Survival analysis of ABCA8 in the UALCAN database. (b) Survival analysis of ABCA8 in the Kaplan-Meier plotter database. [file 13046_2020_1591_MOESM2_ESM.tif]
